# Renal Dysfunction in Chronic Obstructive Pulmonary Disease Lung–Kidney Interorgan Crosstalk with Cardiac Mediation

**DOI:** 10.3390/ijms27072996

**Published:** 2026-03-25

**Authors:** Robert Dragu

**Affiliations:** 1 Internal Medicine C Department, Galilee Medical Center, P.O. Box 21, Nahariya 2210001, Israel; robertd@gmc.gov.il or dragu.msg@gmail.com; Tel.: +972-50-7887795; Fax: +972-4-9107104; 2 Pulmonary Vascular Diseases Service, Galilee Medical Center, Nahariya 2210001, Israel

**Keywords:** chronic obstructive pulmonary disease (COPD), lung–kidney interorgan crosstalk, acute kidney injury, chronic kidney disease, hypoxia inducible factors, endothelial dysfunction, venous congestion, AKI–CKD transition

## Abstract

Chronic obstructive pulmonary disease (COPD) is increasingly recognized as a systemic disorder with clinically significant extrapulmonary manifestations. Among these, renal dysfunction—manifesting as chronic kidney disease (CKD) and acute kidney injury (AKI)—is highly prevalent, frequently underdiagnosed, and strongly associated with adverse clinical outcomes. Meta-analytic data indicate that COPD is associated with more than a twofold increase in CKD prevalence, independent of shared risk factors such as age, smoking, hypertension, and diabetes. CKD in COPD is associated with increased mortality, exacerbation burden, and healthcare utilization. AKI represents a particularly severe expression of renal involvement, occurring most commonly during acute exacerbations of COPD (AECOPD). Although the reported incidence of AKI during AECOPD varies widely by clinical setting—from approximately 2% in population-based studies to over 20% in hospitalized cohorts—its presence is consistently associated with marked increases in mortality, respiratory failure, need for mechanical ventilation, and hospital length of stay. This review synthesizes current evidence supporting a lung–kidney interorgan crosstalk framework in COPD, whereby chronic and acute pulmonary pathophysiology generates systemic disturbances that progressively impair renal structure and function. The heart is incorporated as a physiological intermediary, modulating hemodynamic transmission and venous congestion, without constituting the primary disease axis. Recognizing the role of kidney complications in COPD is crucial, as it influences how we diagnose, predict outcomes, and treat patients—especially when there are sudden flare-ups.

## 1. Introduction

COPD remains a leading cause of global morbidity and mortality and is now widely recognized as a systemic disease rather than a condition confined to the airways. Contemporary management frameworks emphasize the importance of comorbidities in determining prognosis, symptom burden, and treatment response [1]. Among these comorbidities, renal dysfunction has received comparatively limited attention despite mounting evidence of its clinical relevance.

Epidemiological studies consistently demonstrate a higher prevalence of CKD in patients with COPD compared with non-COPD populations [2]. In a large meta-analysis, Gaddam et al. [3] reported that COPD was associated with a 2.2-fold increase in the odds of CKD, even after adjustment for major confounders. More recent systematic reviews confirm this association and further demonstrate that CKD significantly worsens survival in COPD [4]. Longitudinal cohort data indicate that CKD independently predicts mortality in COPD, with hazard ratios exceeding 4.0 in advanced CKD stages [5].

AKI further compounds this burden, particularly during AECOPD. Although AKI may be underrecognized in routine pulmonary practice, its occurrence during exacerbations is associated with dramatic increases in in-hospital mortality and respiratory complications [6,7,8,9,10].

These observations suggest that renal dysfunction in COPD is not incidental but reflects a pathophysiological meaningful interaction between the lung and the kidney. This review adopts a lung–kidney interorgan crosstalk perspective, proposing that pulmonary disease initiates systemic signals—hemodynamic, inflammatory, metabolic, and neurohormonal—that converge on the kidney. The heart is considered an intermediary that modulates hemodynamic transmission and venous congestion, particularly in the presence of pulmonary hypertension and right ventricular dysfunction [11,12].

## 2. Conceptual Framework: Lung–Kidney Interorgan Crosstalk

Interorgan crosstalk refers to dynamic interactions between organ systems mediated by circulating factors, neural pathways, and hemodynamic coupling. In chronic disease states, these interactions frequently evolve from adaptive responses into maladaptive cycles that promote progressive organ injury.

In COPD, the lung constitutes the initiating organ. Chronic airflow limitation, ventilation–perfusion mismatch, impaired alveolar ventilation, and recurrent exacerbations result in sustained hypoxemia, episodic hypercapnia, and systemic inflammatory activation. These pulmonary derangements exert downstream effects on renal perfusion, oxygenation, and cellular homeostasis.

The intermediary role of the heart in lung–kidney interaction has been articulated by Husain-Syed et al., who emphasized that pulmonary disease can influence renal function through hemodynamic and neurohormonal pathways without requiring primary cardiac pathology [11]. Although pulmonary pathology represents the initiating driver in COPD-associated renal injury, right ventricular dysfunction and venous congestion frequently amplify renal stress [12] through hemodynamic transmission. In this framework, the heart acts as a physiological amplifier within the lung–kidney axis rather than an independent initiating organ. In COPD, increased pulmonary vascular resistance, altered intrathoracic pressures, right ventricular loading conditions, and sympathetic activation modify renal blood flow and venous drainage, thereby enhancing the transmission of pulmonary-derived stress signals to the kidney.

Importantly, this framework maintains a lung-centric perspective. Renal injury is conceptualized as a downstream consequence of pulmonary disease severity and instability, rather than as part of a primary cardio–renal syndrome. This distinction is critical to avoid conceptual redundancy while accurately representing physiological mediation.

**Figure 1:**
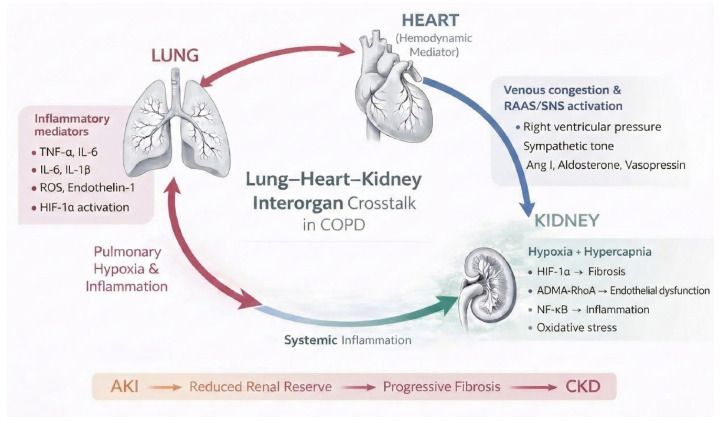
Circular model illustrating how pulmonary hypoxia and inflammation are amplified by cardiac hemodynamic and neurohormonal responses, leading to renal injury and progression from AKI to CKD, with systemic inflammation reinforcing the feedback loop.

The integrated lung–heart–kidney framework and the principal molecular signaling hierarchies underlying this interorgan crosstalk are summarized in Figure 1.

## 3. Chronic COPD as a Driver of Renal Vulnerability

### 3.1. Prevalence and Prognostic Impact of CKD in Stable COPD

Chronic kidney disease is increasingly recognized as a prevalent and clinically meaningful comorbidity in patients with stable COPD. Prevalence estimates vary widely across studies, reflecting differences in population characteristics, comorbidity burden, and—critically—the method used to assess renal function.

Early observational data demonstrated substantial under-recognition of CKD in COPD. In a cohort from Western Norway, Gjerde et al. reported that 31% of COPD patients met criteria for CKD using creatinine-based eGFR, compared with 8% of non-COPD controls, highlighting a significant burden of previously undiagnosed renal impairment [2]. Although this study relied exclusively on creatinine-based equations, it provided important initial evidence that renal dysfunction is common in COPD and frequently overlooked in routine care.

More recent evidence has substantially expanded and refined these observations. A systematic review and meta-analysis [4] including over one million individuals across multiple cohorts, demonstrated that COPD was associated with a more than twofold increased odds of CKD compared with non-COPD populations, even after adjustment for age, smoking, hypertension, and diabetes. This finding supports an association that extends beyond shared risk factors and suggests a disease-specific contribution of COPD-related pathophysiology to renal dysfunction.

Importantly, contemporary studies have emphasized that CKD in COPD is not merely a coincidental comorbidity but carries significant prognostic implications. In a large population-based cohort, Hsu et al. [5] reported that COPD patients with CKD had significantly higher all-cause mortality compared with those without CKD, independent of baseline lung function and cardiovascular comorbidities. The presence of CKD was also associated with increased hospitalization rates and accelerated decline in pulmonary function, underscoring its role as a disease modifier rather than a passive coexisting condition.

Methodological advances over the past decade have also clarified why CKD prevalence may be underestimated in COPD populations. Studies directly comparing creatinine- and cystatin C–based estimates of GFR in COPD, such as Yoshizawa et al. [13] have shown that reliance on creatinine alone may fail to identify a substantial proportion of patients with impaired renal function, particularly in those with sarcopenia or low muscle mass—a common phenotype in advanced COPD. These observations align with contemporary nephrology guidance recommending cystatin C–based assessment when creatinine is likely to be unreliable [14].

Collectively, modern epidemiological evidence indicates that CKD is common, frequently underdiagnosed, and independently associated with adverse outcomes in stable COPD. These findings provide a strong rationale for systematic renal assessment and for incorporating kidney function into COPD risk stratification frameworks.

Sarcopenia is a frequent systemic manifestation of advanced COPD and reflects progressive loss of skeletal muscle mass and function. Chronic systemic inflammation, particularly signalling mediated by tumor necrosis factor-α (TNF-α) and interleukin-6 (IL-6)—together with reduced physical activity and nutritional imbalance—contributes to accelerated proteolysis in skeletal muscle. At the molecular level, activation of the ubiquitin–proteasome pathway and enhanced myostatin signalling play central roles in the pathogenesis of COPD-associated muscle wasting [15].

Beyond its functional consequences, sarcopenia introduces a critical diagnostic limitation in the assessment of renal function. Because serum creatinine originates from skeletal muscle metabolism, reduced muscle mass may produce deceptively low creatinine concentrations despite substantial reductions in true glomerular filtration rate (GFR). Consequently, commonly used creatinine-based equations such as MDRD or CKD-EPI may systematically overestimate renal function in sarcopenic patients. This discrepancy creates a diagnostic “creatinine blind spot,” potentially delaying recognition of chronic kidney disease (CKD) and exposing patients to inappropriate dosing of nephrotoxic medications [13,16].

### 3.2. Pathophysiological Mechanisms Linking Chronic COPD to Kidney Injury

#### 3.2.1. Chronic Hypoxemia and Renal Tissue Hypoxia

Persistent hypoxemia is a defining feature of advanced COPD and a central contributor to renal vulnerability [17]. Renal tissue hypoxia is increasingly recognized as a unifying mechanism driving CKD progression [18]. Experimental and translational studies summarized by Haase and by Wang et al. [19,20] demonstrate that chronic hypoxia induces peritubular capillary rarefaction, promoting fibroblast activation, and irreversible tubulointerstitial fibrosis.

The renal medulla normally operates at low oxygen tension (approximately 10–20 mmHg), rendering it particularly sensitive to reductions in oxygen delivery [18,21]. In COPD, sustained reductions in arterial oxygen content may therefore precipitate tubular hypoxia, impair mitochondrial oxidative phosphorylation, and promote maladaptive repair responses that progressively reduce renal functional reserve.

The master regulator of this response is the Hypoxia-Inducible Factor (HIF) system, specifically HIF-1α in tubular cells and HIF-2α in interstitial and endothelial cells. Under normoxia, prolyl hydroxylase domain (PHD) enzymes hydroxylate HIF-α subunits, targeting them for proteasomal degradation via the von Hippel–Lindau (vHL) tumor suppressor. However, during chronic hypoxemia, stabilized HIF-1α translocates to the nucleus and activates hypoxia-responsive elements (HREs) to initiate transcription of adaptive genes like VEGF and EPO [18,22].

However, a critical “molecular switch” occurs through synergistic crosstalk with the transforming growth factor-beta (TGF-β)/Smad3 pathway. Long-term hypoxia enhances TGF-β signaling, while TGF-β1 treatment reciprocally increases HIF-1α protein expression under normoxic conditions by enhancing its translation rather than its stability. This interdependence is mediated by the ALK5 (TGF-β Type I receptor) kinase activity, which is essential for TGF-β1-stimulated, but not hypoxia-induced, HIF-1α expression. Together, HIF-1α and Smad3 cooperate at the promoter level to drive the expression of Type I collagen, transforming an adaptive survival response into a persistent fibrogenic program [21,23].

In addition to the HIF axis, hypoxia elicits both rapid and sustained activation of the Nuclear Factor-kappa B (NF-κB) pathway, which occurs independently of the conventional inflammatory degradation of IκBα. This pathway is equally effective in promoting a pro-inflammatory transcriptional program within renal tubular epithelial cells [21]. This pathway may promote amplification of the inflammatory milieu and transition to irreversible tubulointerstitial fibrosis [21].

#### 3.2.2. Hypercapnia and Renal Hemodynamic Stress

Chronic and intermittent hypercapnia is prevalent in severe COPD, particularly in patients with chronic respiratory failure or during sleep and exacerbations. Human physiological studies demonstrate that even mild hypercapnia induces renal vasoconstriction and increases renovascular resistance, supporting the concept that CO_2_ retention can impose renal hemodynamic stress [24]. This CO_2_-mediated vasoconstriction is largely driven by a sharp increase in sympathetic tone, characterized by elevated circulating levels of norepinephrine, which results in a dose-dependent reduction in kidney blood flow and impairment of glomerular filtration [17].

In addition, respiratory acidosis increases tubular bicarbonate reabsorption, raising renal oxygen consumption, which in the context of pulmonary-driven hypoxemia exacerbates medullary hypoxia and promotes a state of persistent energy deficit that triggers maladaptive repair responses [25,26,27].

Hypercapnia destabilizes kidney function by indirectly affecting the cardiovascular system [11,17]. Acute rises in CO2 increase pulmonary vascular resistance, causing hemodynamic modifications responsible for reductions in the pressure gradient across glomeruli, finally raising the renal interstitial pressure. At the molecular level, congestion and acidosis can disrupt eNOS activity, leading to superoxide production and oxidative stress that damages endothelium and promotes irreversible tubulointerstitial fibrosis.

#### 3.2.3. Systemic Inflammation and Oxidative Stress

The progression of renal dysfunction in patients with chronic respiratory disease is increasingly attributed to a “systemic spillover” of pulmonary-derived inflammatory mediators into the circulation [17]. Chronic obstructive pulmonary disease (COPD) is characterized by a localized inflammatory response involving the recruitment of neutrophils and macrophages, which release a potent cocktail of pro-inflammatory cytokines, including tumor necrosis factor-alpha (TNF-α), interleukin-6 (IL-6), and interleukin-8 (IL-8) [28,29]. These mediators enter the systemic circulation, where they activate the vascular endothelium and promote a pro-thrombotic, pro-inflammatory state that directly affects renal perfusion and glomerular integrity.

Central to this systemic inflammatory milieu is the activation of the NF-κB signalling pathway. Under conditions of chronic hypoxemia and pulmonary inflammation, hypoxia-inducible factors (HIFs) and Toll-like receptors (TLRs) interact to promote the activation of NF-κB. This transcription factor regulates the expression of genes associated with cellular stress responses and facilitates leukocyte recruitment to the renal interstitium [27,28]. This pathway acts as a key link, where cytokines produced in the lungs initiate a maladaptive inflammatory response in the kidney, leading to tubulointerstitial fibrosis and podocyte damage [14,20,27,29].

Oxidative stress, driven by smoking exposure, inflammation, and hypoxia–reoxygenation cycles, further damages renal endothelial and tubular cells. Vaziri et al. [30] identified oxidative stress as a central mechanism linking chronic inflammation to CKD progression through nitric oxide depletion and profibrotic signaling. Meta-analytic evidence supports inflammation as a mechanistic bridge between COPD and CKD risk [4]. The overproduction of reactive oxygen species (ROS), such as superoxide anions and hydrogen peroxide [22] exert direct cytotoxic effects on renal tubular epithelial cells and scavenge NO, leading to the formation of peroxynitrite. This process significantly impairs renal vasodilation and induces endothelial dysfunction—a hallmark of both COPD and progressive chronic kidney disease [18,21,22,28].

#### 3.2.4. Endothelial Dysfunction and Albuminuria: The ADMA–RhoA Signalling Axis

Endothelial dysfunction represents a central pathophysiological link between chronic pulmonary disease and renal microvascular injury. In COPD, impaired endothelial homeostasis is primarily driven by reduced nitric oxide (NO) bioavailability, reflecting both diminished endothelial nitric oxide synthase (eNOS) activity and increased oxidative stress [17,22,28]. Circulating markers of NO dysregulation, particularly asymmetric dimethylarginine (ADMA), are consistently elevated in COPD and have been shown to discriminate stable disease from acute exacerbations with high diagnostic accuracy [31]. ADMA acts as a competitive endogenous inhibitor of eNOS and simultaneously promotes diversion of L-arginine metabolism toward arginase-dependent pathways, thereby favoring vascular remodeling and endothelial stiffness [32].

The intracellular concentration of ADMA is tightly regulated by dimethylarginine dimethylaminohydrolases (DDAHs). Although both DDAH1 and DDAH2 are expressed in pulmonary and vascular tissues, DDAH1 constitutes the dominant enzymatic route for ADMA degradation, exhibiting substantially higher catalytic efficiency [33,34]. Suppression of DDAH1 activity during chronic hypoxic and inflammatory stress leads to intracellular ADMA accumulation. Importantly, this accumulation not only inhibits NO synthesis but induces eNOS uncoupling, shifting the enzyme from NO production toward superoxide and peroxynitrite generation. This redox imbalance amplifies endothelial oxidative injury and disrupts vasoprotective signaling.

The translation of systemic endothelial dysfunction into renal microvascular injury is mediated through a specific signaling cascade involving Rho-family GTPases. Under physiological conditions, NO-dependent activation of protein kinase G (PKG) maintains endothelial barrier integrity by phosphorylating RhoA at Ser188 [35], thereby suppressing its activity. Elevated ADMA levels attenuate this inhibitory phosphorylation, resulting in sustained RhoA activation. Enhanced RhoA signaling promotes actin stress fiber formation, junctional destabilization, and endothelial-to-mesenchymal transition (EndMT), collectively compromising endothelial barrier permeability [34,35].

Within the renal microcirculation, these molecular events manifest as increased glomerular endothelial leakiness and albuminuria, a finding reported in approximately 20–30% of patients with COPD [36]. Albuminuria in this context should not be viewed merely as a renal comorbidity but rather as a marker of systemic microvascular dysfunction [28,36] reflecting shared pulmonary–renal endothelial pathology. Persistent activation of the ADMA–RhoA axis establishes a feed-forward loop in which oxidative stress further suppresses DDAH1 expression and reduces L-arginine availability, perpetuating endothelial injury and progressive loss of renal functional reserve.

Counter-regulatory mechanisms, including SIRT1- and Nrf2-dependent upregulation of DDAH1, normally serve to mitigate ADMA accumulation and limit mitochondrial reactive oxygen species (ROS)–mediated apoptosis [18,37]. However, during severe or recurrent exacerbations of COPD, these protective pathways become overwhelmed. The resulting imbalance favors sustained endothelial dysfunction, reinforcing pulmonary–renal crosstalk and accelerating microvascular damage across organ systems [34,38].

#### 3.2.5. Neurohormonal Activation and Congestive Renal Stress: The Hemodynamic–Molecular Interface

In advanced chronic obstructive pulmonary disease (COPD), chronic hypoxemia and episodic hypercapnia induce a maladaptive neurohormonal response that primarily alters renal perfusion and venous outflow, thereby establishing the hemodynamic substrate upon which endothelial and molecular injury mechanisms act [17,18,39]. In contrast to the endothelial signaling pathways described in Section 3.2.4, this subsection focuses on the macrocirculatory and neurohormonal drivers of renal stress that precede and amplify microvascular dysfunction.

Sustained gas-exchange abnormalities reduce effective arterial blood volume and activate compensatory neurohormonal systems, including the sympathetic nervous system (SNS), the renin–angiotensin–aldosterone system (RAAS), and arginine vasopressin (AVP) [17,39,40]. While initially adaptive, chronic activation of these pathways leads to persistent renal vasoconstriction, sodium and water retention, and heightened vulnerability to acute kidney injury during pulmonary exacerbations.

Hypoxia- and hypercapnia-mediated chemoreflex activation results in sustained sympathetic overdrive [17,39], reflected by elevated circulating norepinephrine levels in advanced COPD. Increased renal sympathetic nerve activity raises renovascular resistance through afferent and efferent arteriolar vasoconstriction, limiting renal blood flow and constraining renal functional reserve even in the absence of systemic hypotension. Importantly, this sympathetic vasoconstriction acts upstream of the endothelial dysfunction described previously, sensitizing the renal microcirculation to subsequent molecular injury.

In parallel, reduced renal perfusion pressure stimulates renin release and RAAS activation. Angiotensin II–mediated efferent arteriolar constriction transiently preserves glomerular filtration but chronically promotes intraglomerular hypertension, sodium retention, and fibrotic remodeling [17]. Aldosterone further exacerbates distal sodium reabsorption, contributing to volume expansion and venous congestion. This process is compounded by non-osmotic AVP release, driven by sympathetic activation rather than true hyperosmolality, which increases collecting duct water permeability and promotes disproportionate free-water retention.

In COPD patients complicated by pulmonary hypertension or right ventricular (RV) dysfunction, renal injury increasingly reflects venous congestion rather than arterial hypoperfusion. Acute elevations in pulmonary vascular resistance impose excessive RV afterload, resulting in RV dilation and a rise in central venous pressure (CVP). Elevated CVP is transmitted retrogradely to the renal veins, producing renal venous hypertension [11,41,42].

Renal venous congestion directly reduces the transglomerular filtration pressure gradient by increasing Bowman’s capsule pressure, thereby impairing glomerular filtration independently of systemic arterial pressure [11,12]. This mechanism explains the frequent dissociation between preserved systemic hemodynamics and declining renal function observed in congestive states.

At the tissue level, sustained venous congestion initiates a process conceptualized as renal compartment syndrome [11,12]. Due to the kidney’s limited capsular compliance, interstitial edema leads to a rapid rise in intrarenal pressure, compressing peritubular capillaries and aggravating medullary hypoxia [11,17]. Notably, this hypoxic injury is mechanistically distinct from the endothelial barrier failure described in Section 3.2.4, yet synergistic with it.

Medullary hypoxia impairs mitochondrial oxidative phosphorylation, resulting in ATP depletion and functional downregulation of energy-dependent tubular transporters, including the Na^+^/K^+^-ATPase [18,20,21]. This establishes a state of tubular transport failure and metabolic inflexibility, reinforcing sodium retention and perpetuating neurohormonal activation. Over time, these processes promote inflammation, fibrosis, and irreversible nephron loss, facilitating progression from acute kidney injury to chronic kidney disease [12].

The principal molecular signaling hierarchies and pathophysiological switches through which chronic pulmonary disease propagates renal injury are summarized in Table 1 and schematically integrated in Figure 1.

Overview of principal signaling axes linking pulmonary dysfunction to renal injury, including hypoxia (HIF-1α/TGF-β), inflammation (NF-κB), endothelial dysfunction (ADMA–RhoA), and neurohormonal–hemodynamic pathways. Major triggers, mediators, and renal effects are summarized.

### 3.3. Diagnostic Implications: Under Recognition of CKD in COPD

Creatinine-based estimation of glomerular filtration rate (eGFR) systematically underestimates renal dysfunction in chronic obstructive pulmonary disease (COPD). In advanced disease, chronic hypoxemia, systemic inflammation, and prolonged corticosteroid exposure promote skeletal muscle wasting, resulting in reduced endogenous creatinine generation. Consequently, serum creatinine concentrations may remain low despite underlying renal injury, leading to consistent overestimation of eGFR and delayed recognition of chronic kidney disease (CKD) [17]. Failure to recognize occult CKD in COPD has direct clinical consequences, including inappropriate drug dosing, increased susceptibility to contrast-associated kidney injury, and heightened risk of adverse outcomes during acute exacerbations.

Sarcopenia affects approximately 30–40% of patients with advanced COPD and represents a major metabolic determinant of this diagnostic bias. As previously discussed, loss of skeletal muscle mass reduces creatinine production and may obscure early declines in renal filtration capacity. Under these conditions, creatinine-based equations such as MDRD or CKD-EPI lack sensitivity for detecting early renal impairment, particularly when kidney injury is driven by microvascular dysfunction, impaired renal reserve, or tubular bioenergetic stress rather than overt nephron loss [17,43]. The clinical relevance of this limitation was demonstrated in the Norwegian cohort study by Gjerde et al., in which nearly 40% of COPD patients classified as having preserved renal function using creatinine-based equations were reclassified as having CKD when cystatin C–based estimates were applied [2]. Female sex, advanced age, and low body mass index independently predicted discordance between creatinine- and cystatin C–derived GFR, highlighting phenotype-dependent misclassification.

To overcome these limitations, muscle-independent biomarkers have been increasingly proposed. Among them, cystatin C has emerged as a particularly useful biomarker of renal function in populations characterized by altered body composition. Cystatin C is a low-molecular-weight protein produced at a constant rate by all nucleated cells and freely filtered by the glomerulus. Unlike creatinine, its circulating concentration is largely independent of skeletal muscle mass, age, or sex, providing a more reliable estimate of filtration capacity in sarcopenic patients [44].

Clinical studies comparing creatinine- and cystatin C–based GFR estimates in COPD demonstrate that reliance on creatinine alone may fail to identify a substantial proportion of patients with renal impairment. In a cohort study by Yoshizawa et al., a considerable fraction of COPD patients classified as having preserved renal function using creatinine-based equations were reclassified as having CKD when cystatin C–derived estimates were applied [13]. These findings suggest that cystatin C may improve detection of subclinical renal dysfunction and reveal early stages of renal vulnerability within the lung–kidney axis.

Beyond its diagnostic value, cystatin C may also reflect broader systemic processes relevant to COPD. Elevated cystatin C levels have been associated with chronic hypoxemia, systemic inflammation, and endothelial dysfunction, suggesting that this biomarker may capture early renal cellular stress occurring before measurable declines in conventional eGFR [20,43]. Incorporation of these biomarkers enables earlier detection of subclinical renal vulnerability, including congestive renal stress and microvascular injury, thereby improving characterization of the AKI-to-CKD transition. Consistent with this rationale, the 2024 KDIGO guidelines recommend integration of cystatin C when precise GFR estimation is required to guide therapeutic decisions in high-risk populations [14].

### 3.4. Phenotypic Clusters of Increased Renal Risk

Renal impairment in COPD exhibits considerable clinical heterogeneity. Rather than representing a uniform systemic consequence of chronic lung disease, renal vulnerability appears concentrated within specific clinical phenotypes in which pulmonary, hemodynamic, and inflammatory mechanisms converge to impose stress on renal tissues. These interactions extend beyond shared epidemiological risk factors such as aging or tobacco exposure. Instead, emerging evidence suggests that discrete biological endotypes—characterized by persistent systemic inflammation, altered hemodynamics, and metabolic dysregulation—selectively compromise renal filtration and repair capacity in susceptible individuals.

#### 3.4.1. Congestive Bronchitic Phenotype

Patients with chronic bronchitis and recurrent exacerbations frequently develop venous congestion due to increased intrathoracic pressure and impaired right-sided cardiac function. Elevated venous pressure reduces the trans-glomerular filtration gradient and promotes renal interstitial edema, thereby impairing peritubular capillary perfusion and tubular oxygen delivery.

#### 3.4.2. Hypercapnic–Hypoxemic Phenotype

Patients with chronic respiratory failure may exhibit sustained carbon dioxide retention and hypoxemia. Hypercapnia activates both the sympathetic nervous system and the renin–angiotensin–aldosterone system, producing renal vasoconstriction and a reduction in renal blood flow [45].

#### 3.4.3. Sarcopenic-Frailty Phenotype

In patients with systemic frailty and muscle wasting, the diagnostic limitations of creatinine become particularly pronounced. Inflammatory biomarkers such as C-reactive protein and fibrinogen are frequently elevated in this phenotype and have been associated with both skeletal muscle loss and progressive renal injury [46].

#### 3.4.4. Pulmonary Hypertension–Right Ventricular Dysfunction Phenotype

In patients with pulmonary hypertension and right ventricular impairment, reduced cardiac output combined with elevated central venous pressure may lead to congestive nephropathy. In this setting, venous back-pressure exceeds the kidney’s autoregulatory capacity, resulting in tubular hypoxia despite preserved arterial oxygenation [12,47].

### 3.5. Renal Functional Reserve and Renal Fragility

Static measurements of GFR provide only a partial representation of renal physiology. The concept of renal functional reserve (RFR)—defined as the capacity of the kidney to increase its filtration rate in response to physiological stimuli—offers a dynamic measure of renal resilience. RFR reflects the recruitment of previously underutilized nephrons and represents an important indicator of renal adaptability [48,49].

In patients with COPD, chronic hypoxemia and persistent systemic inflammation may progressively diminish this reserve despite apparently preserved baseline GFR. At the cellular level, mitochondrial dysfunction within proximal tubular cells and activation of pro-senescent signaling pathways, including expression of the cell-cycle inhibitor p16INK4a, appear to precede overt structural kidney injury [50].

During acute exacerbations of COPD (AECOPD), this reduced adaptive capacity may predispose the kidney to acute injury when confronted with additional hemodynamic or inflammatory stressors. The resulting “second-hit” scenario can precipitate rapid transition from compensated renal function to acute kidney injury (AKI) [51]. In this context, emerging urinary biomarkers of tubular stress—including insulin-like growth factor–binding protein 7 (IGFBP7) and tissue inhibitor of metalloproteinases-2 (TIMP-2)—may identify early cell-cycle arrest before measurable reductions in GFR occur [52].

## 4. Acute Exacerbations of COPD as a Renal Stress Test

AECOPD represents periods of abrupt systemic destabilization during which pre-existing renal vulnerability frequently manifests as acute kidney injury (AKI). Epidemiologic studies consistently demonstrate that the incidence of AKI is substantially higher during exacerbations than during stable disease, supporting the concept of AECOPD as a critical threshold-crossing event within the lung–kidney axis rather than an isolated pulmonary complication [7,8].

In population-based and hospital cohorts, AKI has been reported in approximately 15–25% of patients hospitalized for AECOPD, with incidence rising further in the presence of advanced age, baseline renal impairment, pulmonary hypertension, or cardiovascular comorbidity [6]. Importantly, Barakat et al. [7] showed that AKI during AECOPD is independently associated with increased short-term mortality, prolonged hospitalization, and incomplete renal recovery, underscoring its clinical relevance beyond transient biochemical changes. Wan et al. [8] further demonstrated that AKI occurring during exacerbations confers a sustained adverse prognostic impact, including increased risk of subsequent exacerbations and long-term mortality.

From a pathophysiological perspective, AECOPD amplifies pre-existing hemodynamic, metabolic, and inflammatory stressors that operate at subclinical levels during stable disease. Acute worsening of hypoxemia and hypercapnia exacerbates renal oxygen supply–demand imbalance [45], precipitating tubular bioenergetic failure characterized by impaired mitochondrial oxidative phosphorylation and reduced Na^+^/K^+^-ATPase activity. This acute metabolic stress occurs on a background of diminished renal reserve, particularly in patients with sarcopenia-associated overestimation of baseline eGFR.

Cardiovascular mediation further determines renal outcomes during exacerbations. Acute increases in pulmonary vascular resistance and right ventricular dysfunction elevate central venous pressure, which is transmitted retrogradely to the renal venous system [11,41,53]. The resulting reduction in the transglomerular filtration pressure gradient, together with venous congestion–induced interstitial edema, promotes a functional renal compartment syndrome and exacerbates medullary hypoxia.

At the molecular level, the convergence of hypoxic, mechanical, and inflammatory stress during AECOPD activates injury-sustaining signaling programs that impair renal recovery. Acute renal hypoxia induces rapid NF-κB activation through calcium-dependent and TAK1–IKK–mediated pathways, while concurrent stabilization of HIF-1α and engagement of TGF-β/Smad3 signaling favors persistence of inflammatory and profibrotic transcriptional responses. These mechanisms provide a biological explanation for the high rate of incomplete renal recovery and the observed transition from AKI to chronic kidney disease following AECOPD.

### AKI-to-CKD Transition

Acute kidney injury complicating acute exacerbations of chronic obstructive pulmonary disease (AECOPD) constitutes a critical node in the AKI–acute kidney disease–chronic kidney disease (AKI–AKD–CKD) continuum. Although serum creatinine often normalizes with respiratory and hemodynamic stabilization, growing evidence indicates that AECOPD unmasks a renal vulnerability marked by incomplete structural recovery and accelerated long-term nephron loss.

Renal recovery after AECOPD depends on the resolution of pathological lung–kidney crosstalk. Correction of hypercapnia and hypoxemia reduces pulmonary vascular resistance and right-sided filling pressures, alleviating renal venous congestion and restoring the transglomerular filtration gradient. However, this hemodynamic improvement frequently occurs within a persistent inflammatory and neurohormonal milieu. Sustained activation of profibrotic pathways, including transforming growth factor-β and Wnt/β-catenin signaling [54], promotes maladaptive tubular repair, interstitial fibrosis, and transition toward chronic kidney disease despite apparent biochemical recovery.

Longitudinal clinical data support this paradigm. Patients with a hospitalized AECOPD phenotype—characterized by a substantially increased risk of AKI—exhibit a significantly steeper long-term decline in estimated glomerular filtration rate compared with COPD patients without hospitalized exacerbations (approximately −4.6 vs. −2.4 mL/min/1.73 m^2^/year) [44]. These findings suggest that severe exacerbations act as recurrent renal insults, accelerating CKD progression even when overt AKI episodes are clinically transient. Importantly, stabilization of serum creatinine in the post-exacerbation period may therefore obscure ongoing subclinical nephron loss during the AKD window (7–90 days), a phase increasingly recognized as decisive for long-term renal outcomes.

Although COPD-specific studies directly quantifying post-AECOPD AKI eGFR trajectories remain limited, extrapolation from broader AKI literature supports a cumulative, dose–response relationship between recurrent kidney insults and CKD progression. Within this framework, AECOPD-associated AKI should be viewed not as an isolated complication, but as a mechanistic driver of chronic renal disease evolution. These observations underscore the need for systematic renal surveillance following AECOPD, even in patients with apparent short-term recovery. Emerging biomarkers of tubular stress such as neutrophil gelatinase-associated lipocalin (NGAL), kidney injury molecule-1 (KIM-1), and the cell-cycle arrest markers TIMP-2 and IGFBP7 may enable earlier identification of maladaptive repair processes following AECOPD-associated AKI [5,52,55].

## 5. Clinical Implications: Toward Kidney-Aware COPD Management

Recognition of lung–kidney crosstalk has direct clinical implications. Given the high prevalence and prognostic impact of renal dysfunction, systematic renal risk stratification should be integrated into COPD care.

In stable disease, assessment should include eGFR and albuminuria, with selective use of cystatin C in frail or sarcopenic patients. During AECOPD, AKI prevention should be considered a core management goal, including early renal function assessment, avoidance of nephrotoxins, individualized fluid management, and recognition of congestion-related renal stress.

Patients experiencing AKI during AECOPD should undergo structured post-discharge renal follow-up, as incomplete recovery is common and predicts subsequent CKD progression.

Situations warranting nephrology consultation include persistent decline in eGFR following AECOPD-associated AKI, unexplained albuminuria, or recurrent exacerbation-associated renal injury. Early nephrology involvement may facilitate risk stratification, optimization of nephroprotective strategies, and monitoring for AKI–CKD transition.

## 6. Key Takeaways and Future Directions

- Renal dysfunction represents an underrecognized systemic manifestation of COPD.
- Sarcopenia may obscure renal dysfunction when creatinine-based estimation is used.
- Acute exacerbations of COPD function as renal stress tests that may accelerate AKI–CKD transition.

Future work should prioritize refined lung–kidney phenotyping in COPD, identification of biomarkers signaling renal vulnerability, and targeted interventions to interrupt maladaptive interorgan crosstalk. Systematic incorporation of renal endpoints into COPD clinical trials will be essential to enable earlier risk stratification and accelerate translation into practice.

## 7. Conclusions

This review integrates molecular hypoxia signaling, endothelial dysfunction, and venous congestion into a unified lung–kidney crosstalk framework specific to COPD.

Renal dysfunction is a common yet underrecognized and prognostically relevant component of COPD. Both chronic and acute kidney injury arise from lung–kidney interorgan crosstalk driven by pulmonary pathophysiology, with cardiac involvement modulating hemodynamic transmission rather than defining the primary disease axis. Integrating kidney-aware strategies into COPD management offers a pragmatic route to improved outcomes and reflects a broader transition toward systems-based care in chronic respiratory disease.

## Figures and Tables

**Table 1:** Molecular Signaling Hierarchies and Pathophysiological Switches Orchestrating Lung–Kidney Interorgan Crosstalk.

Signaling Pathway or Mechanism	Molecular Mediators and Switches	Physiological Trigger	Renal Impact and Cellular Effect	Key Regulatory Proteins	Crosstalk Result
HIF-1α/TGF-β/Smad3 Crosstalk	ALK5 receptor signaling and PHD-dependent HIF stabilization	Chronic hypoxemia	Peritubular capillary rarefaction, fibroblast activation, and enhanced extracellular matrix deposition	HIF-1α, Smad3, and vHL tumor suppressor	Progressive tubulointerstitial fibrosis with limited reversibility
NF-*k*B Signaling Pathway	I*k*Bα degradation and Toll-like receptors signaling	Sustained systemic inflammation and chronic hypoxemia	Leukocyte recruitment to the renal interstitium and activation of pro-inflammatory transcriptional program	NF-κB, hypoxia-inducible factors (HIFs), and TAK1–IKK signaling complex	Tubulointerstitial fibrosis and podocyte injury
ADMA–RhoA Signaling Axis	Impaired dimethylarginine dimethylaminohydrolase (DDAH) activity and RhoA activation	Reduced nitric oxide bioavailability and oxidative stress	Endothelial-to-mesenchymal transition (EndMT) and junctional destabilization	Asymmetric dimethylarginine (ADMA), RhoA, endothelial nitric oxide synthase (eNOS), and protein kinase G (PKG)	Endothelial dysfunction and albuminuria
Neurohormonal–Hemodynamic Interface	Na^+^/K^+^-ATPase and Arginine Vasopressin (AVP)	Hypercapnia and venous congestion	Renal vasoconstriction, reduced transglomerular filtration gradient, and interstitial edema	Norepinephrine, Angiotensin II, and Aldosterone	Renal compartment syndrome and functional renal reserve decline
Oxidative Stress Pathway	Excess generation of reactive oxygen species, including superoxide anions and hydrogen peroxide	Hypoxia–reoxygenation cycles	Peroxynitrite formation and scavenging of Nitric Oxide (NO)	Uncoupled endothelial nitric oxide synthase (eNOS), SIRT1, and Nrf2	Endothelial dysfunction and renal tubular epithelial cell cytotoxicity

## Data Availability

No new data were created or analyzed in this study. Data sharing is not applicable to this article.
